# Creativity and productivity during the COVID-19 pandemic

**DOI:** 10.1038/s41598-023-40493-y

**Published:** 2023-09-05

**Authors:** Yvonne Görlich

**Affiliations:** Department of Psychology, PFH Private University of Applied Science Göttingen, 37073 Göttingen, Germany

**Keywords:** Psychology, Health care

## Abstract

This study explored impacts of the COVID-19 pandemic on creativity and productivity and how personality variables moderated these impacts. Two online self-report surveys were conducted. 863 (spring 2020) and 421 (spring 2021) participants were asked how the corona crisis affected their creativity and productivity. In addition, personality variables, namely the Big Five (openness, conscientiousness, extraversion, agreeableness, neuroticism), as well as interpersonal trust, need for cognition, risk-taking, and life satisfaction, were assessed. As a result of the crisis, the group of participants appeared more creative in 2020, while no significant group effect of the pandemic was found for productivity. In 2021, however, the crisis had a negative impact on creativity and productivity. In 2020, predictors for an improved creativity were openness, conscientiousness, neuroticism, and life satisfaction; predictors for improved productivity were conscientiousness, negative interpersonal trust, and life satisfaction. In 2021, only life satisfaction predicted improved creativity, while improved productivity was predicted by conscientiousness, negative neuroticism, and life satisfaction. At its beginning, the COVID-19 pandemic had, on average, a positive effect on creativity and a neutral one on productivity. Later, the impact turned negative on both creativity and productivity. Here, lower life satisfaction was particularly relevant.

## Introduction

The COVID-19 pandemic, with its contact restrictions and lockdowns, has affected the daily lives of nearly everyone. Many people have been studying and working from home, suffering from a lack of childcare. Social leisure activities have been severely restricted. One might assume that restrictions on personal freedom can be demotivating and impede creativity and productivity^1,2^. On the other hand, crises provide opportunities and challenges for creativity, necessitate and accelerate transitions in work processes^3^. Illuminating those opposing effects is thus a timely task for psychological research, in particular from a crisis management perspective.

Various surveys have revealed an increase in adverse psychological symptoms such as anxiety, depression, and stress during the COVID-19 crisis^4–6^. Chinese studies during the COVID-19 pandemic showed positive correlations between negative mood and creativity^7,8^. Engaging in creative tasks can potentially buffer the negative effects of living through the pandemic^9^. One study reported significant positive correlations between creativity and death reflections, while there was little or no negative correlation between creativity and death anxiety^10^.

Creativity is defined as the discovery of novel and useful products and ideas^11,12^. Other authors^13,14^ added surprising as a third point to this definition: This is about experiencing the "Aha!" or eureka moment. Creativity can be subdivided into everyday creativity (little c) and eminent creativity (big c)^15^. Creativity is relevant in various domains, e.g., science, engineering, music, art, crafts, humour, literature, architecture, mechanics, cooking, sports, dance, drama, invention, performance, and (information) technology. Productivity can be measured as performance at work or at school, as a subjective self-evaluation or an evaluation by others (e.g., teachers, supervisors), or by more objective criteria like sales figures, the output of academic publications, or more globally as Gross Domestic Product. Productivity is about the production of products, concrete results, and performance.

Studies on the impact of the COVID-19 pandemic on creativity and productivity have produced differential results. One study did not see any significant differences in professional creativity before and during the lockdown but everyday creativity appeared significantly increased during lockdown with a small effect (*d* = 0.15)^16^. Another study, conducted from May to June 2020, reported significant correlations with a small effect between the perceived impact of COVID-19 and creativity^17^. Teachers mostly reported that the pandemic had limited their options for providing creative opportunities to students^18^. Another study revealed that researchers in the field of radiation oncology felt less productive during the pandemic^19^. A survey of software developers, conducted at two-week intervals during the Covid-19 pandemic, revealed no productivity differences to the pre-pandemic situation^20^.

Another study showed that children had higher originality scores and came up with more ideas when seated separately from classmates^21^. Thus, social distancing necessitated by the COVID-19 pandemic can positively influence creativity, especially in people who like to be alone.

Creativity and productivity may be related to social interactions, environmental factors, and personality traits. The five-factor model of personality (Big Five) distinguishes openness to experience, conscientiousness, extraversion, agreeableness, and neuroticism^22^. Above all, openness^23–25^ and extraversion^26,27^ are associated with creativity. Productivity, job and academic performance are related to conscientiousness^28–30^.

Interpersonal trust can be relevant for performance^31^ and creativity^32^. Also, the need for cognition^33^,^34^ is positively associated with academic performance (Elias and Loomis, 2002, #6125},^35^, and creativity or innovative behaviour^36–38^.

The average life satisfaction is related to productivity in 20 European countries^39^. Another study showed that well-being is associated with less productivity loss^40^. For creativity, a meta-analysis found an effect of r = 0.14 with well-being, which is associated with life satisfaction^41^. With a focus on the COVID-19 pandemic, one study found a medium correlation between self-reported creativity and well-being^17^.

For risk-taking, studies have found a positive correlation with creativity^42,43^ and performance^44^. Creative risk-taking can be a moderator of creative outcomes in crises^45^. Dynamics and the uncertainty of COVID-19 necessitate creative and innovative solutions^46^.

Studies during the COVID-19 pandemic showed that especially the personality trait neuroticism was significantly positively associated with perceived stress, depression, and loneliness^47–49^ and negatively with well-being^50^.

The crisis led to creative solutions to specific problems of the COVID-19 pandemic, such as measures for reducing exposure to the coronavirus at the onset of the crisis, the design of medical ventilators, or pharmaceutical companies repurposing existing drugs (like Remdesivir or Molnupiravir) to treat COVID-19 symptoms^46^.

Contacts in pandemic times were often only virtual; working from home allowed for more time flexibility, and saved time otherwise spent for commuting. The cessation of many leisure activities should have left more time for and reduced interference with productivity and creativity.

Flexible working conditions or online groups can increase productivity^51^ and creativity^52^. In virtual teams, creativity is found to be positively correlated with the establishment of rapport and participation equality, and negatively correlated with conflict^53^. It has been shown experimentally that working from home can improve performance^54^. However, in an interview during the 2020 Covid-19 pandemic, the author of this study also suggested that being forced to work from home while teaching and caring for children was not conducive to performance^55^. He analysed the impact of Covid-19 on business productivity and found that the factor productivity (TFP) fell by up to 5% during 2020–2021^56^.

The present study took the pandemic as a unique opportunity to study the impacts of a crisis situation, crisis-related changes in working conditions and restrictions on personal freedom, on creativity, performance and productivity. It was also a unique opportunity to study personal traits as modifiers of a crisis response. As the crisis lasted for quite a long period of time, with new virus variants and challenges emerging, this also invited to study these crisis effects over time.

## Methods

### Study design

Two online surveys were conducted via LimeSurvey in Germany, exploring impacts of the Corona crisis on creativity and considering personal traits as modifiers. The samples were convenience samples, acquired through the author and students. The first survey took place during the first lockdown in spring 2020, from 30th March to 4th June 2020, the second during the second lockdown in spring 2021, from 11th April 2021 to 25th May 2021. During the lockdowns in Germany, most stores were closed, students’ lessons were mostly held online, working from home was made possible and encouraged, and strict contact restrictions were enforced, wearing masks in public was mandatory.

### Sample

A total of 1284 participants took part in the study: 863 in spring 2020 and 421 in spring 2021. The description of the samples can be found in Table 1. The age ranged from 18 to 91 (*M* = 33.01; *SD* = 14.89) in spring 2020 and from 18 to 85 (*M* = 33.76; *SD* = 15.99) in spring 2021. Between the two samples were no significant differences in age, gender or mother tongue (see Table 2) as well as in highest school-leaving qualification (Mann–Whitney-*U*-test: *p* = 0.399).

**Table 1:** Sample characteristics.

	Spring 2020		Spring 2021	
	*N*	Percent (%)	*N*	Percent (%)
Gender				
Female	543	62.9	262	62.2
Male	310	35.9	154	36.6
Diverse	2	0.2	3	0.7
Not specified	8	0.9	2	0.5
Mother tongue				
German	814	94.3	404	96.0
Non-German	39	4.5	16	3.8
Not specified	10	1.2	1	0.2
Highest school-leaving qualification				
A-level/high school diploma	594	68.8	299	71.0
Vocational baccalaureate	106	12.3	58	13.8
Intermediate school-leaving qualification	133	15.4	52	12.4
Lower secondary school-leaving qualification	24	2.8	8	1.9
No secondary school-leaving qualification	1	0.1	4	1.0
Not specified	5	0.6	0	0
Current occupation (multiple answers possible)				
Working people	465	53.9	189	44.9
University student	316	36.6	181	43.0
Vocational student	48	5.6	28	6.7
Pupil	24	2.8	20	4.8
Housewife/-men/parental leave	32	3.7	14	3.3
Retired	26	3.0	14	3.3
Unemployed	17	2.0	9	2.1
Not specified	13	1.5	3	0.7

**Table 2:** T-tests comparing both measurement times.

	Spring 2020			Spring 2021			Differences						
	*N*	*M*	*SD*	*N*	*M*	*SD*	*M*	*T*	*df*	*p* (two-tailed)	*CI* under	*CI* upper	*d*
Impact on creativity	863	4.33	1.14	421	3.78	1.30	0.55	7.74	1282	< 0.001	0.41	0.69	0.45
Impact on productivity	863	3.91	1.29	421	3.57	1.33	0.34	4.46	1282	< 0.001	0.19	0.50	0.26
Openness	863	3.63	1.03	421	3.71	1.01	− 0.08	− 1.28	1282	0.202	− 0.20	0.04	0.08
Conscientiousness	863	3.65	0.83	421	3.57	0.86	0.08	1.57	1282	0.116	− 0.02	0.18	0.09
Extraversion	863	3.41	1.01	421	3.43	0.99	− 0.01	− 0.18	1282	0.855	− 0.13	0.11	0.01
Agreeableness	863	3.28	0.84	421	3.26	0.72	0.02	0.37	1282	0.714	− 0.08	0.11	0.02
Neuroticism	863	2.91	0.96	421	2.98	0.93	− 0.07	− 1.32	1282	0.186	− 0.19	0.04	0.08
Interpersonal trust	863	3.53	0.78	421	3.48	0.75	0.05	1.13	1282	0.260	− 0.04	0.14	0.07
Need for cognition	863	4.88	1.05	421	4.77	1.03	0.12	1.91	1282	0.056	0.00	0.24	0.11
Risk taking	863	4.18	1.41	421	4.24	1.46	− 0.07	− 0.78	1282	0.434	− 0.23	0.10	0.05
Life satisfaction	863	5.10	1.29	421	4.87	1.34	0.23	2.95	1282	0.003	0.08	0.38	0.17
Age	856	33.01	14.89	420	33.76	15.99	− 0.75	− 0.83	1274	0.409	− 2.53	1.03	0.05
Gender (1 = female, 2 = male)	853	1.36	0.48	416	1.37	0.48	− 0.01	− 0.24	1267	0.814	− 0.06	0.05	0.02
Mother tongue (1 = German, 2 = non-German)	853	1.05	0.21	420	1.04	0.19	0.01	0.63	1271	0.530	− 0.02	0.03	0.05

*Note* At both measurement times range of given answers was from 1 to 7 for impact on creativity, impact on productivity, risk-taking, and life satisfaction minimum, from 1 to 5 for openness, conscientiousness, extraversion, agreeableness, and neuroticism, from 1 (2020) resp. 1.33 (2021) to 5 for interpersonal trust, and from 1.6 (2020) resp. 2 (2021) to 7 for need for cognition.

### Measurements

Single items were used to evaluate the impact of the Corona pandemic on creativity and productivity. The following questions were asked: Has the Corona crisis had an impact on your creativity? Response options on a 7-step scale: 1 = I am much less creative; 2 = I am less creative; 3 = I am rather less creative; 4 = no impact; 5 = I am rather more creative; 6 = I am more creative; 7 = I am much more creative. Has the Corona crisis had an impact on your productivity? Response options on a 7-step scale: 1 = I am much less productive; 2 = I am less productive; 3 = I am rather less productive; 4 = no impact; 5 = I am rather more productive; 6 = I am more productive; 7 = I am much more productive. After each question, the answer could be detailed in a free text field: What is the reason for this? How does it show?

BFI-10^57^ was used to measure the personality factors openness, conscientiousness, extraversion, agreeableness, and neuroticism. BFI-10 is a ten-item-scale (each factor is measured via 2 items) with response options on a 5-step scale from 1 = disagree strongly to 5 = agree strongly; retest-reliability after 6 to 8 weeks were for openness 0.72, for conscientiousness 0.77, extraversion 0.83, for agreeableness 0.68, and for neuroticism 0.74^57^.

The construct of interpersonal trust was measured via KUSIV3^58^, a three-item-scale with response options on a 5-step scale from 1 = don’t agree at all to 5 = agree completely with a retest reliability (interval of 6 to 10 weeks) of 0.57 and an McDonadls Omega of 0.85. Cronbach’s Alpha, calculated in the present study, were in sample 1 (spring 2020) 0.78 and in sample 2 (spring 2021) 0.79.

Need for cognition (NFC) was assessed by NFC-K-2^59^, a five-item scale with response options on a 7-step scale from 1 = does not apply at all to 7 = fully applies. Cronbach´s alpha was 0.69^59^, in this current study, Cronbach´s Alpha was for sample 1 (spring 2020) 0.73 and for sample 2 (spring 2021) 0.69.

Risk-taking was measured via R-1^60^, a single-item scale with response options on a 7-step scale from 1 = not at all willing to take risks to 7 = very willing to take risks. The retest reliability after 6 weeks was 0.74.

Life satisfaction was measured via L-1^61^, a single item scale with response options on a 7-step scale from 1 = not at all satisfied to 7 = completely satisfied; the retest reliability after 6 weeks was 0.67.

For all scales with more than one item, the mean of the items was the total value.

In addition, demographic questions were asked about age, gender, school-leaving qualification, and current occupation.

### Statistical procedures

Data were analysed with IBM SPSS Statistics (version 25). Mean value comparisons were performed by *t*-test for one sample and for independent samples. In the one sample t-test, the value 4 = no impact was used as the reference value for comparison. Effect sizes are given as Cohen’s *d*^62^. Correlation analyses were calculated as Pearson correlations (*r*). Dichotomous variables were assigned dummy codes. Regression analyses were calculated in a linear fashion. Cronbach’s *α* was calculated for each survey scale with more than 2 items. The open questions on the justification were summarized in terms of content.

### Ethics

All persons participated in the surveys voluntarily and anonymously, they gave written informed consent. The study was approved by the ethics committee of the PFH Private University of Applied Sciences Göttingen, followed the ethical guidelines of the PFH Private University of Applied Sciences Göttingen and was in accordance with the guidelines of the German Psychology Association (DGPs).

## Results

### Impact of the COVID-19 pandemic on creativity

**Figure 1:**
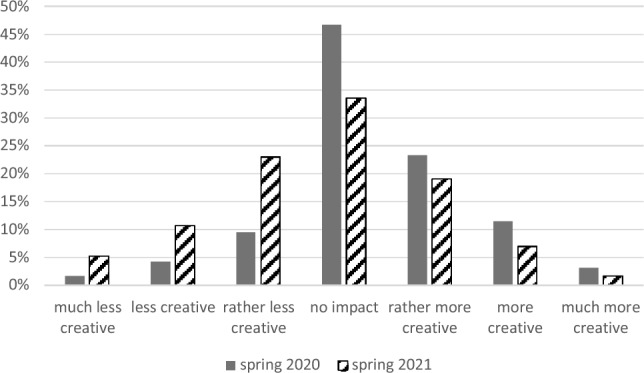
Impact of the Corona crises on creativity.

In spring 2020, respondents indicated a significant increase in their creativity as a result of the Corona Crisis (one sample *t*-test: mean difference = 0.33; *SD* = 1.14;* t* = 8.43; *df* = 862; *p* < 0.001; 95% *CI* 0.25; 0.40; *d* = 0.29). The detailed response pattern is shown in Fig. 1: 38% of respondents described themselves as more creative (ranging from rather to much more creative) as a result of the crisis. 15% experienced themselves as less creative (ranging from rather less to much less creative) and 47% saw no impact of the Corona Crisis on their creativity. By contrast, in spring 2021, a significant decrease in creativity (one sample t-test: mean difference = 0.22; *SD* = 1.30;* t* = − 3.52; *df* = 420; *p* < 0.001; 95% *CI* − 0.35; − 0.10; *d* = 0.17) was evaluated. This time, no impact on their creativity was seen by 33%, 28% experienced themselves as more creative (ranging from rather to much more creative) and 39% as less creative (ranging from rather to much less creative).

#### Reasons for more creativity during the COVID-19 pandemic

In 2020 and 2021, a very frequently cited reason for more creativity during the Corona crisis was more available time. Other reasons included: fewer distractions, new problems to solve (more mentions 2020), own biorhythms, active corona research, better focus, better ideas due to rest, more balance, and finding new ways/alternatives.

#### Reasons for less creativity during the COVID-19 pandemic

Individuals who perceived themselves as less creative as a result of the crisis cited the following reasons: Double burden of work and childcare/homeschooling (2020), unstructured everyday life, different daily routine, distractions, less inspiration due to lockdown, missing impressions from the outside world, exchange with colleagues and creative meetings were missing, always being in the same place leading to fewer ideas, lack of motivation, monotony, stress, tension, more thoughts, worries, uncertainty, uncertain future, irritability, listlessness, laziness, boredom, dissatisfaction with the situation, lack of concentration, restriction of freedom, compulsion to stay home and existential fears. In 2021, depression and actual COVID-19 infections were added.

### Impact of the COVID-19 pandemic on productivity

**Figure 2:**
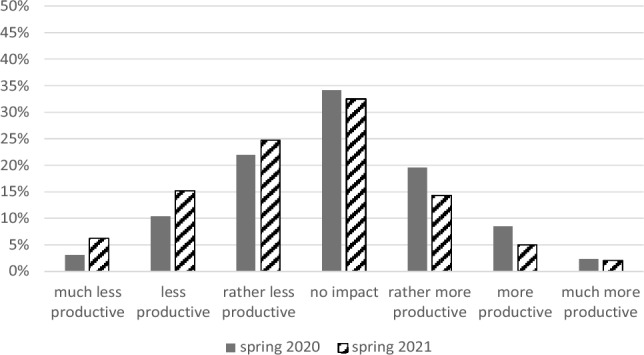
Impact of the Corona crises on productivity.

In 2020, there was no significant change in productivity because of the Corona crisis (one sample t-test: mean difference = − 0.09; *SD* = 1.29;* t* = − 1.96; *df* = 862; *p* = 0.050; 95% *CI* − 0.17; 0.00; *d* = 0.07). In 2021, however, a significant decrease in productivity (one sample t-test: mean difference = 0.43; *SD* = 1.33; *t* = − 6.64; *df* = 420; *p* < 0.001; 95% *CI* − 0.56; − 0.30; *d* = 0.32) was found. Figure 2 shows the pattern of responses: 35% (2020) resp. 46% (2021) felt at least "rather less productive" as a result of the crisis, and 31% (2020) resp. 21% (2021) as at least rather more productive, 34% (2020) resp. 33% (2021) of respondents saw no impact of the Corona crisis on their productivity.

#### Reasons for more productivity during the COVID-19 pandemic

The following reasons were cited for being more productive during the crisis: more available time, free time management, enjoyment of working or learning from home (2020), no need to travel, working focused from home, getting unfinished things done (2020), fewer distractions or other commitments, working hours adaptable to individual daily and sleep rhythms, better focus due to fewer private appointments, being well-rested, no pressure, less exhaustion, better project management due to digital solutions, working in healthcare, to-do-lists (2021), more turnover, more customers (2021).

#### Reasons for less productivity during the COVID-19 pandemic

People who experience themselves as less productive gave the following reasons: no daily structure, lack of drive, no motivation, lack of social contacts, no workplace at home (2020), distraction, lack of orders, lack of pressure, incompatibility of working from home and childcare (2020), poorer working or studying from home. In 2021 the following were added: lack of variety, depression, no vacation, listlessness, stress, fatigue, lack of compensation, less concentration, too much work.

### Mean differences between 2020 and 2021

Compared with 2020, participants in 2021 indicated that the Corona crisis had a negative impact on their creativity (*d* = 0.45, see Table 2) and productivity (*d* = 0.26). These differences were significant. Otherwise, only life satisfaction (*d* = 0.17) showed a significant difference between the two samples. There were no significant differences between the groups for openness, conscientiousness, extraversion, agreeableness, neuroticism, interpersonal trust, need for cognition, risk-taking, age, gender and mother tongue (for details, see Table 2).

For an interpretation of the mean values of the variables examined, these can be compared with values from reference samples. For the Big Five^63^, interpersonal trust^58^ and risk-taking^60^, these are available from a random sample of 1134 participants from the year 2010. These are for openness *M* = 3.41, *SD* = 0.93, for conscientiousness *M* = 4.15, *SD* = 0.79; for extraversion *M* = 3.47, *SD* = 0.95; agreeableness *M* = 3.45, *SD* = 0.95, neuroticism *M* = 2.42, *SD* = 0.88, interpersonal trust *M* = 3.37, *SD* = 0.77, risk taking *M* = 3. 61, *SD* = 1.59. This means that the participants in this current study showed slightly higher scores in openness, neuroticism, interpersonal trust and risk-taking and slightly lower scores in conscientiousness and agreeableness than the reference group. Life satisfaction at measurement time 1 is comparable to the data from a quota sample (*M* = 5.05, *SD* = 1.23, N = 407)^61^, need for cognition in a reference sample was *M* = 5.22, *SD* = 1.03^59^, so in the study presented here the values are somewhat lower.

### Correlation analyses

The correlation of the assessment of the increase in creativity and productivity due to the Corona crisis with other personality variables is shown in Table 3. In 2020, significant but small correlations with the increase in creativity due to the Corona crisis were found with openness (*r* = 0.17, *p* < 0.001), conscientiousness, extraversion, agreeableness (each *r* = 0.09, *p* = 0.006 resp. *p* = 0.008), and life satisfaction (*r* = 0.11, *p* = 0.002). In 2021, only conscientiousness (*r* = 0.10,* p* = 0.032), life satisfaction (*r* = 0.17, *p* < 0.001) and age (*r* = 0.11, *p* = 0.027) correlated with the increase in creativity due to the Corona crisis.

**Table 3:** Intercorrelation matrix.

	1	2	3	4	5	6	7	8	9	10	11	12	13	14
1 Impact on creativity		0.435**	0.030	0.104*	− 0.032	0.012	− 0.035	0.050	0.009	0.046	0.172**	0.108*	0.051	− 0.023
2 Impact on productivity	0.421**		− 0.003	0.276**	− 0.003	0.017	− 0.161**	0.110*	0.096	− 0.021	0.244**	0.201**	0.071	− 0.048
3 Openness	0.165**	0.031		0.108*	0.105*	0.083	0.059	0.099*	0.219**	0.100*	0.007	0.025	− 0.198**	0.057
4 Conscientiousness	0.093**	0.203**	− 0.026		0.135**	0.128**	− 0.125*	0.151**	0.268**	− 0.021	0.260**	0.221**	− 0.163**	− 0.054
5 Extraversion	0.093**	0.091**	0.143**	0.073*		0.067	− 0.193**	0.179**	0.056	0.244**	0.204**	− 0.047	− 0.073	0.003
6 Agreeableness	0.091**	0.006	0.076*	0.030	0.094**		0.044	0.401**	0.016	− 0.065	0.081	0.102*	− 0.166**	0.111*
7 Neuroticism	0.020	− 0.090**	− 0.007	− 0.096**	− 0.211**	− 0.045		− 0.151**	− 0.225**	− 0.258**	− 0.238**	− 0.170**	− 0.320**	0.011
8 Interpersonal trust	0.040	− 0.019	0.109**	0.045	0.188**	0.348**	− 0.164**		0.153**	0.040	0.302**	0.253**	− 0.099*	− 0.011
9 Need for cognition	0.008	0.021	0.092**	0.202**	0.021	− 0.002	− 0.126**	0.106**		0.184**	0.248**	0.056	0.059	− 0.015
10 Risk taking	0.063	0.074*	0.085*	0.001	0.240**	0.030	− 0.301**	0.007	0.119**		0.131**	− 0.036	0.201**	0.111*
11 Life satisfaction	0.105**	0.165**	0.013	0.244**	0.221**	0.152**	− 0.301**	0.271**	0.113**	0.146**		0.203**	− 0.018	− 0.056
12 Age	− 0.049	0.018	− 0.028	0.279**	0.022	0.010	− 0.158**	0.121**	− 0.057	− 0.052	0.128**		0.085	− 0.019
13 Gender	− 0.005	− 0.001	− 0.078*	− 0.159**	− 0.049	− 0.142**	− 0.267**	− 0.048	0.118**	0.215**	0.018	0.027		0.007
14 Mother tongue	− 0.037	− 0.055	− 0.032	− 0.012	− 0.042	− 0.078*	0.054	− 0.031	0.010	− 0.059	− 0.099**	0.022	0.019	

*Note* Pearson correlation; intercorrelations below the diagonal from spring 2020 (N = 863, for age N = 856, for gender and mother tongue N = 853) and above the diagonal from spring 2021 (N = 421, for age and mother tongue N = 420, for gender N = 416); gender: 1 = female, 2 = male; mother tongue: 1 = German, 2 = non-German).

*p < 0.05; **p < 0.01.

With the increase in productivity, significant correlations were found with conscientiousness (*r* = 0.20, *p* < 0.001), extraversion (*r* = 0.09, *p* = 0.007), neuroticism (*r* = − 0.09, *p* = 0.008), risk-taking (*r* = 0.07, *p* = 0.030), and life satisfaction (*r* = 0.17, *p* < 0.001) in 2020. While in 2021, conscientiousness (*r* = 0.28, *p* < 0.001), neuroticism (*r* = − 0.16, *p* = 0.001), interpersonal trust (*r* = 0.11, *p* = 0.025), life satisfaction (*r* = 0.24, *p* < 0.001) and age (*r* = 0.20, *p* < 0.001) significantly correlated with the increase in productivity due to the Corona crisis.

The experienced increase in creativity and productivity due to the corona crisis correlated positively with 0.42 (*p* < 0.001) in 2020 and with 0.44 (*p* < 0.001) in 2021; no significant correlations of these variables with age in 2020 and gender in either year were found.

### Multiple regression analyses

Regressions were calculated to account for possible multicollinearities (Table 4). In 2020, personality variables correlated with increases in creativity with *R* = 0.24 (*R*^2^ = 0.06), whereas openness, conscientiousness, neuroticism and life satisfaction showed significant standardized beta weights. In 2021 *R* was 0.22 (*R*^2^ = 0.05), and only life satisfaction had a significant stand. beta weight.

**Table 4:** Regression analysis for impact on creativity and productivity through the COVID 19 pandemic.

	Impact on creativity		Impact on productivity	
	Spring 2020	Spring 2021	Spring 2020	Spring 2021
Standardized beta weights for:				
Openness	0.155**	0.039	0.035	− 0.004
Conscientiousness	0.085*	0.087	0.179**	0.232**
Extraversion	0.048	− 0.096	0.045	− 0.084
Agreeableness	0.068	− 0.009	− 0.001	− 0.034
Neuroticism	0.076*	− 0.004	− 0.031	− 0.117*
Interpersonal trust	− 0.022	0.009	− 0.073*	0.038
Need for cognition	− 0.028	− 0.074	− 0.034	− 0.028
Risk taking	0.050	0.056	0.038	− 0.047
Life satisfaction	0.086*	0.176**	0.120**	0.178**
R	0.239	0.215	0.260	0.359
R^2^	0.057	0.046	0.068	0.129
N	863	421	863	421

*Note* ANOVA of all models are significant (*p* < 0.05); *p < 0.05, **p < 0.01.

Regarding the increase in productivity in 2020, conscientiousness, life satisfaction, and negative interpersonal trust had significant beta weights; the multiple correlation coefficient was *R* = 0.26 (*R*^2^ = 0.07). In spring 2021, *R* was 0.36 (*R*^2^ = 0.13) with significant standardized beta weights for conscientiousness, life satisfaction, and negative neuroticism (emotional stability).

## Discussion

The Corona crisis, and in particular the measures taken to contain it, had a major impact on everyone's lives. This included isolation, shifting work to home offices, distance learning and lack of childcare. Coping with the crisis required problem solving, it spurred technological transitions such as online meetings, and unleashed creative potential.

Indeed, people in the large sample of the present study felt more creative on average at the start of the crisis (in spring 2020). One year later, they felt less creative. The differences between the time points were significant. This difference can be explained by the novelty of the situation in 2020, while in 2021, the then chronic crisis was no longer stimulating but tiring, depressing and demotivating. This is consistent with a decline in productivity from 2020 to 2021. These negative trends on productivity seem to be favoured by neuroticism.

Self-assessment of the impact of the corona crisis on one's own creativity was significantly related to personality structure, although these correlations are rather small. Thus, openness, conscientiousness and neuroticism (when life satisfaction is also considered) had a positive influence on the increase in creativity in spring 2020. This result is interesting and can be related to previous studies^7,8^. People who perceive themselves as more creative may also be more thoughtful, brooding or anxious. However, this is only apparent through the influence of life satisfaction, which had a positive effect on the assessment of the increase in creativity. At the beginning of the pandemic, there were new problems to solve, so respondents experienced a positive effect of the pandemic on their creativity. One year later, the problems caused by the pandemic were no longer new. The fact that life satisfaction was positively related to both increased creativity and productivity may also be related to the effects of psychological stress during the crisis. Studies showed an increase in depression^64,65^, which also implies a decrease in life satisfaction.

One main reason for an experienced increase in creativity and productivity was more available time (which held true only when lack of childcare was not an issue), while a main reason for a decrease in creativity and productivity was a lack of daily structure. How people dealt with the lack of daily structure and uncertainties caused by the crisis is also related to personality structure. The lack of motivation, social isolation or fear reported in the present study were also reasons for the decrease in creativity among Brazilian students due to the pandemic^66^.

The personality trait conscientiousness was positively associated with the increase in productivity due to the crisis in both spring 2020 and spring 2021. This is consistent with the results of a study in which lower situational strength due to COVID-19 was associated with a stronger positive effect of conscientiousness on performance^67^.

The regression analyses did not show an effect of the variables extraversion, agreeableness, need for cognition, and risk-taking for either time point, neither for the influence of the pandemic on creativity nor on productivity.

This study also shows clear individual differences. People who stay at home tend to have less structured days. Some are then more creative and productive as they can organise their time flexibly and according to their individual biorhythms. For others, this is a disadvantage, leading, for example, to more procrastination—a trend observed during the pandemic^68^.

Working conditions at home also played an important role. Parents were often double-burdened with childcare while working, with negative feedback on both, productivity and creativity, while others were able to retreat in peace and use this time for undisturbed creative output. Working from home showed negative correlations between productivity and family-work conflict, social isolation, distracting environment and stress, but positive correlations with job autonomy, self-leadership and work engagement^69^.

It is striking that the same reasons tend to lead to a reduction in productivity and creativity for some people and an increase for others. From this, it can be concluded that individual solutions are to be favoured. Thus, which work and which working conditions lead to more creativity and productivity depends partly on the personality, but also on the preferences of the individual. Working or studying from home is very beneficial for some people, but less so for others; some people need the exchange, while others do not. Individuals who are more disciplined and structured in their work feel that working from home increases productivity and creativity, while individuals who need a daily structure and "pressure" are more likely to be more productive and creative in a collaborative work situation.

This has implications for human resource management: a “new reality” that offers new opportunities to which organizational scholars and practitioners will need and want to remain attentive^70^. Innovations in science persist after the Corona pandemic^71^. Therefore, scientific public interest should be present regardless of a pandemic, as should the consensus that society needs science.

Crises can increase creativity, but rather at the beginning of the crisis and not for everyone. To maintain creativity and productivity over a longer period of crisis, it is important to implement measures (e.g. psychological self-help programmes^72^) that maintain life satisfaction and related well-being.

### Limitations

The limitations of the study are: The two surveys in 2020 and 2021 were conducted as independent cohort studies, so they are not comparing the same individuals. The effects on creativity and productivity were asked directly. If the data had been available before the Covid-19 pandemic, an indirect comparison could have been made. Also, in the present study, the impact of the Corona crisis on creativity and productivity was measured globally via one item each. Further studies could, for example, differentiate by areas of creativity (e.g., domains of creativity^73^ or forms of creativity^15^). In this study, participants tended to refer to the "little c" and everyday creativity. A literature review on the impact of the COVID-19 pandemic on the creative industries showed that some sectors, such as IT and software, benefited from the pandemic, while others, such as festivals and culture events, were negatively affected^74^. In addition, the respective study also pointed out that it is above all the restrictions imposed by political regulations that have an impact on creativity and productivity. This has implications for the design of working and living conditions in future crises. The study was conducted in Germany, and it cannot be ensured whether the results are transferable to other countries.

### Supplementary Information

Supplementary Information.

## Data Availability

All data analysed during this study are included in this published article (see supplementary information file).
